# Overexpression of the Replicative Helicase in *Escherichia coli* Inhibits Replication Initiation and Replication Fork Reloading

**DOI:** 10.1016/j.jmb.2016.01.018

**Published:** 2016-03-27

**Authors:** Jan-Gert Brüning, Kamila Katarzyna Myka, Peter McGlynn

**Affiliations:** Department of Biology, University of York, Wentworth Way, York YO10 5DD, United Kingdom

**Keywords:** ssDNA, single-stranded DNA, DAPI, 4′,6-diamidino-2-phenylindole, DNA replication, DNA repair, genome stability, transcription, disease

## Abstract

Replicative helicases play central roles in chromosome duplication and their assembly onto DNA is regulated via initiators and helicase loader proteins. The *Escherichia coli* replicative helicase DnaB and the helicase loader DnaC form a DnaB_6_–DnaC_6_ complex that is required for loading DnaB onto single-stranded DNA. Overexpression of *dnaC* inhibits replication by promoting continual rebinding of DnaC to DnaB and consequent prevention of helicase translocation. Here we show that overexpression of *dnaB* also inhibits growth and chromosome duplication. This inhibition is countered by co-overexpression of wild-type DnaC but not of a DnaC mutant that cannot interact with DnaB, indicating that a reduction in DnaB_6_–DnaC_6_ concentration is responsible for the phenotypes associated with elevated DnaB concentration. Partial defects in the *oriC-*specific initiator DnaA and in PriA-specific initiation away from *oriC* during replication repair sensitise cells to *dnaB* overexpression. Absence of the accessory replicative helicase Rep, resulting in increased replication blockage and thus increased reinitiation away from *oriC*, also exacerbates DnaB-induced defects. These findings indicate that elevated levels of helicase perturb replication initiation not only at origins of replication but also during fork repair at other sites on the chromosome. Thus, imbalances in levels of the replicative helicase and helicase loader can inhibit replication both via *inhibition* of DnaB_6_–DnaC_6_ complex formation with excess DnaB, as shown here, and *promotion* of formation of DnaB_6_–DnaC_6_ complexes with excess DnaC [Allen GC, Jr., Kornberg A. Fine balance in the regulation of DnaB helicase by DnaC protein in replication in *Escherichia coli*. J. Biol. Chem. 1991;266:22096–22101; Skarstad K, Wold S. The speed of the *Escherichia coli* fork *in vivo* depends on the DnaB:DnaC ratio. Mol. Microbiol. 1995;17:825–831]. Thus, there are two mechanisms by which an imbalance in the replicative helicase and its associated loader protein can inhibit genome duplication.

## Highlights

• Loading of the replicative helicase is the key step in replisome assembly.
• Increasing replicative helicase concentration in *E. coli* inhibits growth.
• Inhibition is due to helicase complexes depleted of the helicase loader protein.
• Depletion inhibits replication initiation and reinitiation during replication repair.
• Imbalances in replicative helicase components can prevent replication initiation.

## Graphical Abstract

**Figure:**
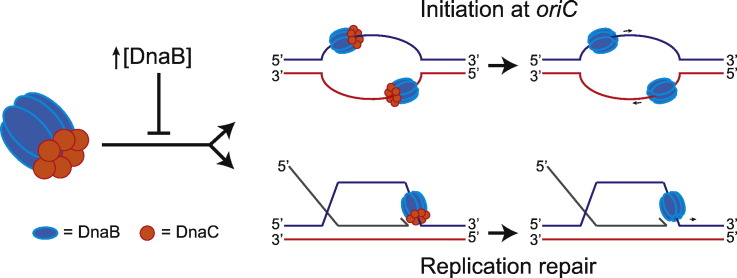

## Introduction

The structural complexity of the genetic material of a cell demands complex copying systems to achieve high-fidelity DNA replication. Replicative helicases are at the leading edge of replication forks, both driving strand separation and acting as a protein interaction hub at the heart of the replisome [3]. These helicases are active as hexamers that form toroidal quaternary structures and bind a single strand of nucleic acid in their central channel [4]. Unwinding of duplex DNA is achieved by NTP-driven translocation along this bound single-stranded DNA (ssDNA) and steric exclusion of the complementary strand creating ssDNA templates that can then be copied by DNA polymerases [5].

The replicative helicase needs to be loaded at an origin of replication before it can begin to unwind DNA. In *Escherichia coli*, the initiation of replication starts with binding of *oriC* by the ATP-bound initiator protein DnaA that leads to melting of the DNA duplex and creation of a ssDNA bubble [6]. Helicase loading also requires the helicase loader protein DnaC in complex with a DnaB hexamer in a 6:6 ratio [7]. During initiation, the circular DnaB hexamer is opened within this complex, allowing the ssDNA to be passed into the central channel of the helicase [8]. ATP hydrolysis by DnaC results in its dissociation, allowing DnaB to translocate along ssDNA towards the fork junction and subsequent association of other enzymes with DnaB to form the active replisome [9]. However, if DnaC is in excess over DnaB, then DnaB helicase and thus replisome activity are inhibited [1], [2]. Inhibition results from the ATP-bound form of DnaC continually reassociating with DnaB to form a DnaB_6_–DnaC_6_ complex that cannot translocate [10], [11].

Once a replication fork is translocating along the template DNA, then potential barriers such as DNA damage or nucleoprotein complexes are encountered frequently [12], [13], [14], [15]. Some of these barriers can be bypassed or cleared by the original replisome, allowing replication to continue [16], [17]. However, some nucleoprotein barriers, especially those associated with transcription, need to be cleared ahead of the fork by accessory replicative helicases such as Rep in *E. coli* [18], [19]. This removal of nucleoprotein barriers minimises replisome pause time and hence reduces the probability of replisome inactivation since replisomes lose activity as a function of pause time [20], [21], [22]. In *E. coli*, this minimisation of nucleoprotein-induced fork pausing by Rep is promoted by a physical interaction between the Rep C-terminus and DnaB [18], [23], [24].

Replisome inactivation still occurs in spite of mechanisms that reduce the probability of loss of replisome function [13], [25]. In such circumstances, the replisome must be reloaded back onto the chromosome in order to complete genome duplication. Replisome reloading is triggered by the reassembly of the replicative helicase back onto the chromosome in a reaction that, as with *oriC*, requires DnaC [26]. However, the presence of the ssDNA binding protein SSB prevents DnaB loading onto ssDNA and replication initiator proteins are need to overcome this inhibition [6]. DnaA-mediated replication initiation is sequence specific and occurs only at the origin, and thus, additional factors are required away from *oriC* to overcome this SSB-dependent barrier. In *E. coli*, two pathways that facilitate reloading of the replisome back onto the chromosome away from *oriC* exist [25], [27]. One pathway for replication fork reloading involves the helicase PriA. PriA binds to DNA forks possessing a 3′ OH group of a nascent leading strand close to the fork branch point in an orientation allowing PriA translocation along the lagging strand template [28], [29]. PriA binding to the fork results in recruitment of the additional reloading factors PriB and DnaT and subsequent loading of DnaB onto the lagging strand template via a DnaB–DnaC dodecamer [30], [31], [32], [33], [34]. Alternatively, DnaB reloading can be catalysed by PriC. PriC has complementary forked DNA binding specificity to that of PriA, targeting forks lacking a 3′ OH group of a nascent strand close to the fork branch point, but the outcome again is replicative helicase reloading onto the lagging strand template [25], [27]. If a ssDNA region is absent on the lagging strand template, additional DNA unwinding by the 3′-to-5′ helicases Rep or PriA is required to provide sufficient ssDNA for PriC-directed DnaB loading [25], [35].

We have found that overexpression of DnaB in *E. coli* has a modest inhibitory effect on chromosome duplication. This inhibition is alleviated upon co-overexpression of the helicase loader DnaC indicating that a reduction in the concentration of DnaB_6_–DnaC_6_ complexes, needed for DnaB loading during replication initiation and reinitiation [7], is responsible for inhibition of chromosome duplication by excess DnaB. Partial defects in DnaA-dependent replication initiation at *oriC* or in PriA-dependent replication reinitiation away from *oriC* act synergistically to increase the toxicity of DnaB overexpression. Therefore, overexpression of the replicative helicase impacts on replisome assembly at and away from *oriC*. However, absence of PriC does not hypersensitise cells to excess DnaB indicating that, in otherwise unstressed cells, PriA-directed fork repair predominates or that PriC-specific substrates can be targeted by PriA either with or without further processing of the forked DNA. The inhibitory effect of excess DnaB is also exacerbated in cells lacking the accessory replicative helicase Rep that correlates with the elevated replication blockage and thus replication reinitiation needed in Δ*rep* cells [19], [36]. These data demonstrate that elevation of replicative helicase levels can result in inhibition of chromosome duplication. Thus, paradoxically, inhibition of replication can occur both by promotion of DnaB_6_–DnaC_6_ complex formation via excess DnaC [1], [2] and by reducing the probability of DnaB_6_–DnaC_6_ complex formation via excess DnaB as shown here. Such imbalances could conceivably result in replicative stress regardless of the organism.

## Results

### Overexpression of DnaB inhibits colony formation and this inhibition is exacerbated by the absence of Rep

**Fig. 1:**
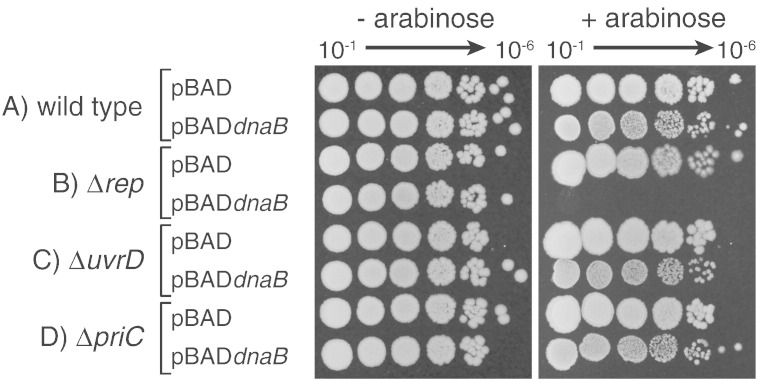
Overexpression of *dnaB* inhibits growth of cells lacking the accessory replicative helicase Rep. Induction of *dnaB* overexpression was performed using a plasmid-based, arabinose-inducible promoter system. Strains harbouring the indicated plasmids were grown in the absence of arabinose and then serial dilutions plated onto LB agar without and with 0.2% arabinose. Wild type, *rep*, *uvrD* and *priC* strains (TB28, N6577, N6632 and MKG3, respectively) were transformed with pBAD or pBAD*dnaB* (pJGB143).

**Fig. 2:**
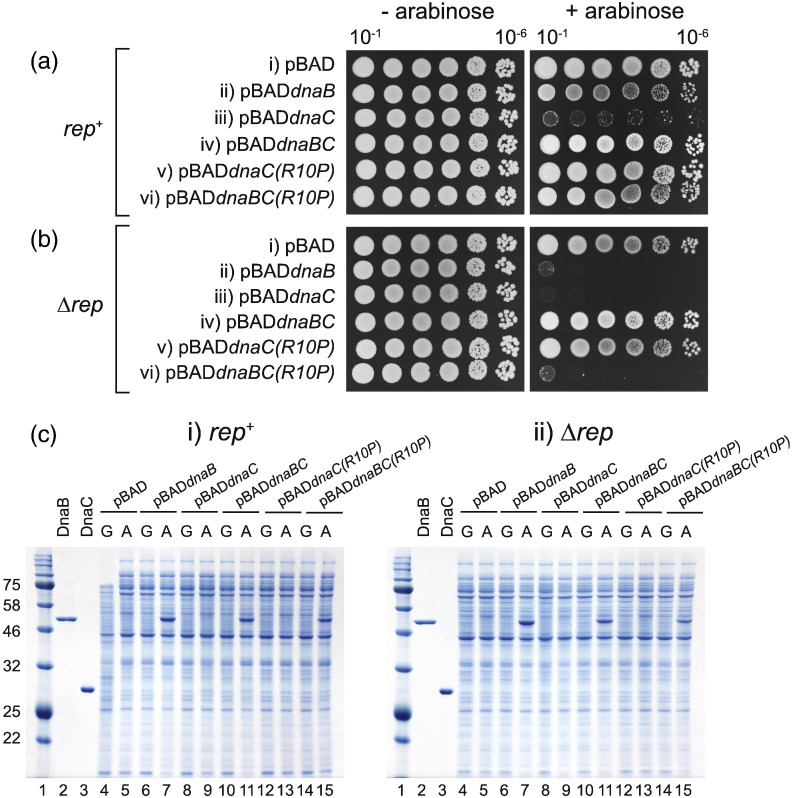
Alleviation of the *dnaB*-induced growth defect by *dnaC*. The colony-forming ability of (a) wild type (TB28) and (b) Δ*rep* (N6577) strains containing the indicated plasmids was analysed on LB agar without and with 0.2% arabinose. Plasmids (i)–(vi) were pBAD, pJGB143, pJGB408, pJGB404, pJGB415 and pJGB418, respectively. (c) Protein content of the strains in (a) and (b) as monitored by SDS polyacrylamide gel electrophoresis. Lane 1 contains molecular mass markers with their apparent molecular mass in kilodaltons (kDa). Lanes 2 and 3 contain 0.4 μg purified DnaB and 0.5 μg purified DnaC, respectively. Lanes 4–15 contain samples from the indicated strains grown in either 0.2% glucose (g) or 0.2% arabinose (a).

As part of ongoing attempts to probe the physical interaction between the primary and the accessory replicative helicases in *E. coli*, we constructed an arabinose-inducible overexpression plasmid encoding *dnaB* that increased intracellular levels of DnaB in the presence of arabinose (Fig. 2c). This induction in a wild-type strain did not reduce the number of colony-forming units but did decrease colony size as compared with a control containing the empty vector pBAD (Fig. 1A). This decrease in colony size suggested that overexpression of the replicative helicase was moderately deleterious. Given the original purpose of the overexpression plasmid, we also tested the impact of *dnaB* overexpression in a strain lacking the gene encoding the accessory replicative helicase in *E. coli*. In contrast to the wild-type strain, overexpression of *dnaB* was extremely inhibitory to colony formation in Δ*rep* cells, resulting in at least a 10^4^-fold decrease in colony-forming ability (Fig. 1B). We conclude that elevated levels of DnaB inhibit growth of an otherwise wild-type strain and that inhibition is greatly exacerbated in the absence of Rep.

We then tested which function of Rep was responsible for hypersensitivity of Δ*rep* cells to *dnaB* overexpression. Rep has been implicated in promoting fork movement along protein-bound DNA and also in promoting PriC-dependent reloading of DnaB [18], [25], [35]. However, Δ*priC* cells displayed only a modest decrease in colony size similar to that seen in wild type (Fig. 1D). Therefore, a defect in PriC-directed replisome reloading was not responsible for the *dnaB* hypersensitivity of Δ*rep* cells. Thus, absence of the only other known function of Rep, promotion of fork movement along protein-bound DNA, may be the missing function that hypersensitises cells to *dnaB* overexpression.

A helicase closely related to Rep, UvrD, promotes replisome movement along protein-bound DNA in the absence of Rep [18], [19]. However, UvrD cannot compensate fully for the absence of Rep [18], [24] resulting in Δ*rep* but not Δ*uvrD* cells displaying increased replisome pausing at nucleoprotein complexes [36]. *dnaB* overexpression in Δ*uvrD* cells again gave only a modest decrease in colony size similar to that seen in the wild type (Fig. 1C). These data indicate that the partial ability of UvrD to compensate for the absence of Rep-promoted fork movement is not sufficient to ameliorate the effects of *dnaB* overexpression.

### *dnaC* counters *dnaB* toxicity

A 1:1 DnaB:DnaC ratio is needed for maximal DnaB helicase and replisome activity [1]. Therefore, we analysed whether the effects of *dnaB* overexpression reflected a substoichiometric level of DnaC with respect to DnaB.

*dnaC* was cloned into the same arabinose-inducible overexpression plasmid as that used for *dnaB*, creating pBAD*dnaC.* Growth of *rep*^+^ and Δ*rep* cells containing pBAD*dnaC* inhibited growth in the presence of arabinose, as expected [1], [2] (Fig. 2a-iii and b-iii). In contrast, pBAD*dnaBC* in which wild-type *dnaC* had been cloned downstream of *dnaB* suppressed the growth defects caused by overexpression of *dnaB*, a suppression that was most apparent in Δ*rep* cells (Fig. 2b, compare ii and iv). Suppression of the *dnaB*-dependent growth defect in pBAD*dnaBC* was not due to lack of elevated DnaB levels as SDS polyacrylamide gel electrophoresis revealed similar levels of DnaB in cells containing pBAD*dnaB* and pBAD*dnaBC* (Fig. 2c, compare lanes 7 and 11 in i and ii). However, we could not detect increased intracellular levels of DnaC with either pBAD*dnaC* or pBAD*dnaBC* (Fig. 2c, compare lanes 9 and 11 in i and ii). This was surprising given the ability of pBAD*dnaC* to inhibit growth, as expected when DnaC concentrations are elevated [1], [2]. It is possible that overexpressed DnaC was degraded rapidly but it is also possible that suppression of DnaB toxicity can be achieved by substoichiometric elevation of DnaC levels. A third formal possibility is that suppression by *dnaC* is not dependent on formation of DnaB–DnaC complexes and is thus not dependent on the DnaB:DnaC ratio, but it occurs via some other unidentified mechanism. This alternative mechanism cannot be absence of DnaB overexpression due to the cloning of *dnaC* downstream of *dnaB* as DnaB was overexpressed from pBAD*dnaBC* clones (Fig. 2c-i and c-ii, lanes 11 and 15). Furthermore, suppression was dependent on the interaction of DnaC with DnaB. DnaC(R10P) is deficient only in DnaB binding and, thus, does not inhibit growth when overexpressed [37] [see also pBAD*dnaC*(*R10P*) in Fig. 2a-v and b-v]. Cloning of *dnaC*(*R10P*) downstream of *dnaB* failed to alleviate inhibition of growth by *dnaB* in Δ*rep* cells (Fig. 2b, compare iv and vi). We conclude that DnaC counters the growth defects associated with elevated DnaB via a DnaB–DnaC interaction.

### Hypersensitivity of Δ*rep* cells to *dnaB* overexpression is alleviated by reducing nucleoprotein barriers to replication

**Fig. 3:**
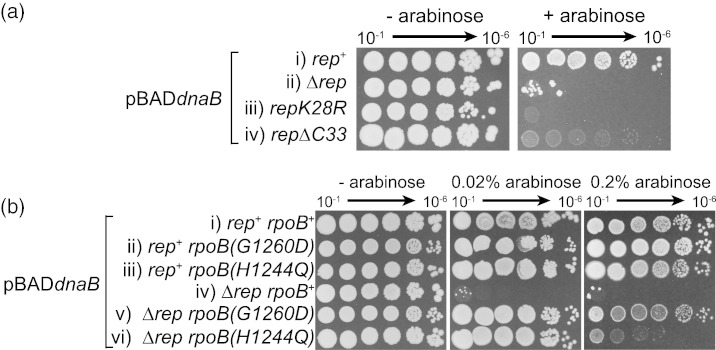
Sensitivity to *dnaB* overexpression correlates with levels of replisome pausing and breakdown. (a) Colony-forming ability of the indicated strains [(i–iv) JGB064, N6577, SS1076 and JGB066, respectively] containing pJGB143 without and with 0.2% arabinose. (b) Colony-forming ability of *rep*^+^ and Δ*rep* strains bearing pJGB143 with the indicated *rpoB* mutations in the presence of 0%, 0.02% and 0.2% arabinose. Strains (i)–(vi) were TB28, AM2158, N5925, N6577, HB278 and N7604, respectively.

Minimisation of replisome pausing and breakdown at nucleoprotein complexes is dependent on Rep helicase activity and is also promoted by a physical interaction between DnaB and the C-terminus of Rep [18], [19], [36]. *repK28R* encodes a helicase-deficient Rep that lacks all accessory helicase function but retains the DnaB interaction domain [24]. Cells bearing *repK28R* displayed hypersensitivity to *dnaB* overexpression similar to that seen in Δ*rep* cells (Fig. 3a-iii). The *rep*Δ*C33* allele encodes Rep that retains helicase activity but lacks the C-terminal DnaB interaction domain, resulting in a reduction in rather than abolition of accessory helicase activity [18], [24]. *dnaB* overexpression in *rep*Δ*C33* cells did not lead to the large reduction in colony numbers seen in Δ*rep* and *repK28R* cells but did confer a large decrease in colony size (Fig. 3a-iv). Thus, Rep helicase function is essential for tolerance of *dnaB* overexpression and the Rep–DnaB interaction promotes this tolerance, a pattern similar to that found for minimisation of replisome pausing by Rep [36].

We also tested whether mutations known to decrease, rather than increase, replisome pausing and breakdown countered sensitivity to *dnaB* overexpression. RNA polymerase mutations that destabilise transcription complexes or inhibit backtracking suppress genome duplication defects by reducing nucleoprotein barriers to replication [14], [36], [38], [39]. We tested two such mutations, *rpoB*(*G1260D*) and *rpoB*(*H1244Q*) [40]. Both *rpo* mutations suppressed the toxicity of *dnaB* overexpression in Δ*rep* cells (Fig. 3b). Suppression of toxicity under high levels of *dnaB* induction was greater for *rpoB*(*G1260D*) than for *rpoB*(*H1244Q*) (Fig. 3b, compare v and vi with iv on 0.2% arabinose), reflecting patterns of suppression of genome duplication defects conferred by these mutations [36], [40]. However, both *rpoB* mutations provided robust suppression at an intermediate arabinose concentration (Fig. 3b, 0.02% arabinose). Therefore, mutations known to reduce replicative barriers suppress the hypersensitivity of Δ*rep* cells to *dnaB* overexpression.

Taken together, these data indicate that inhibition of growth in Δ*rep* cells by *dnaB* overexpression is related to increased frequency of replisome pausing and breakdown at protein–DNA complexes.

### Elevated levels of DnaB inhibit chromosome duplication in Δ*rep* cells

We probed the impact of *dnaB* overexpression upon genome duplication by monitoring chromosome content using flow cytometry under run-out conditions. These conditions allow cells to complete ongoing rounds of replication but prevent reinitiation of replication and inhibit cell division [41]. Such conditions provide an indication of the numbers of origins per cell and the ability of cells to complete chromosome replication during the 2-h course of the run out.

**Fig. 4:**
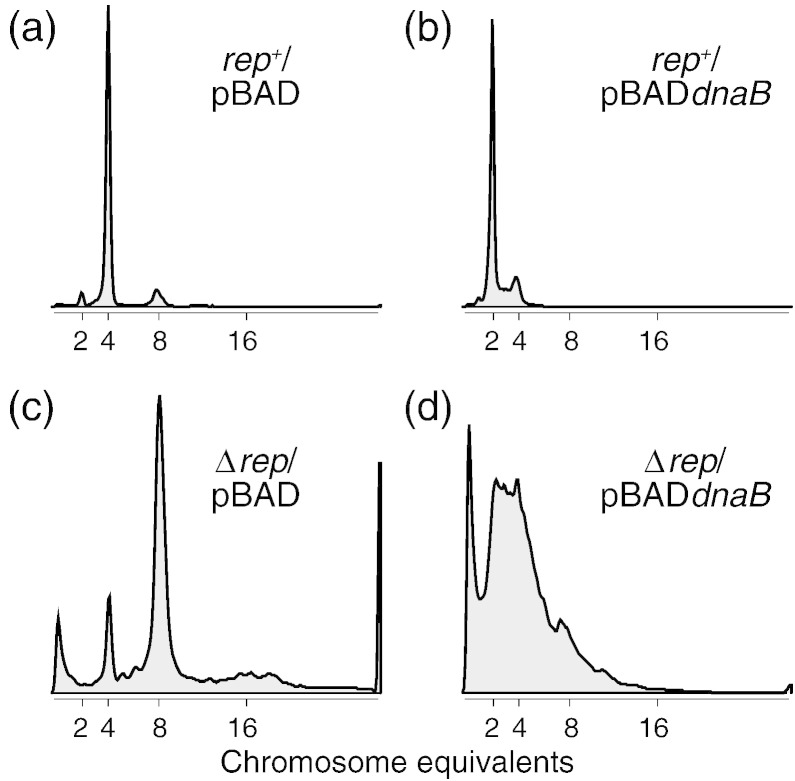
Overexpression of *dnaB* perturbs chromosome replication in wild type and in Δ*rep* cells. Strains were grown in LB in the absence of arabinose and then shifted into LB plus 0.2% arabinose for 2 h. Initiation at *oriC* and cell division were then inhibited for 2 h (“runout conditions”) and DNA content was monitored by flow cytometry. Strains (a) and (b) were TB28 harbouring pBAD and pJGB143, respectively, whilst strains (c) and (d) were N6577 with pBAD and pJGB143.

Overexpression of *dnaB* resulted in a decrease in the median number of chromosome equivalents from 4 to 2 in wild-type cells, indicating that elevated levels of DnaB cause a decrease in *oriC* numbers per cell (Fig. 4, compare a and b). This decrease in the median number of origins per cell suggests some perturbation of replication initiation at *oriC*. However, the majority of cells overexpressing *dnaB* contained an integral number of chromosomes (Fig. 4b). Therefore, chromosome duplication was achieved within the 2-h run out indicating completion of the elongation and termination phases of replication.

In Δ*rep* cells, the number of origins per cell was higher than in *rep*^+^ cells even in the absence of *dnaB* overexpression (Fig. 4, compare a and c). This effect is due to increased chromosome duplication time in Δ*rep* cells that results in more frequent reinitiation prior to termination of replication [24], [36], [42]. Induction of *dnaB* overexpression in Δ*rep* cells caused a major defect in chromosome metabolism evinced by the inability to generate intact, discrete chromosomes (Fig. 4d). Therefore, elevated DnaB concentrations severely inhibit completion of chromosome duplication in the absence of Rep.

**Fig. 5:**
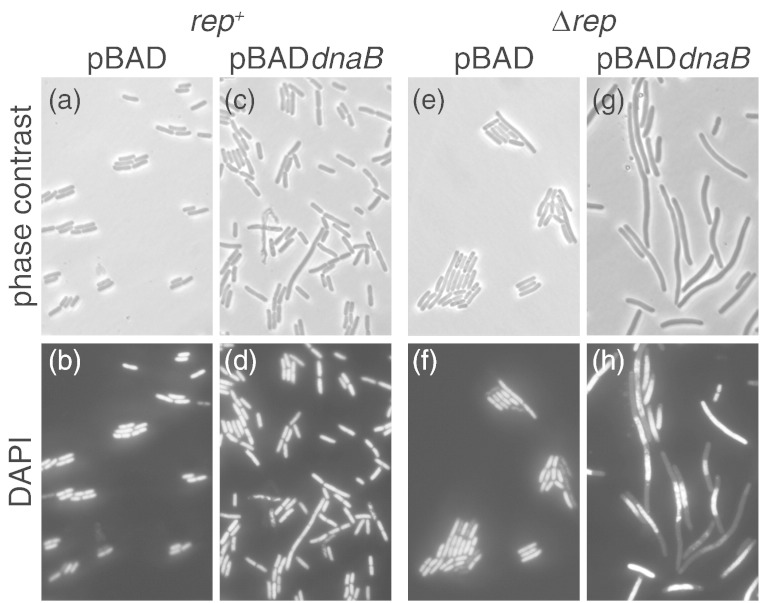
Impact of *dnaB* overexpression on cell and nucleoid morphology. Wild type (TB28) and Δ*rep* cells (N6577) containing either pBAD or pBAD*dnaB* (pJGB143) were grown in LB in the absence of arabinose prior to shifting into LB containing 0.2% arabinose. Strains were then grown to mid-logarithmic phase and analysed by phase contrast microscopy and DAPI staining of nucleoids.

We imaged *rep*^+^ and Δ*rep* cells without and with *dnaB* overexpression. Overexpression in *rep*^+^ cells did not result in significant perturbation of nucleoid structure (Fig. 5, compare a and b with c and d). Occasional chains of cells were observed in the *rep*^+^ strain with elevated DnaB but the nucleoids in these chains appeared similar in structure to those found in the absence of *dnaB* overexpression (Fig. 5, compare b and d). Δ*rep* cells formed occasional elongated cells even in the absence of *dnaB* overexpression but nucleoid structure was similar to that observed in *rep*^+^ cells (Fig. 5, compare a and b and e and f). In contrast, *dnaB* overexpression in Δ*rep* cells resulted in mainly filamentous cells (Fig. 5g and h). Within these filaments, the nucleoids were extended but these filaments also contained significant volumes lacking DNA (Fig. 5g and h), indicative of an inability to complete chromosome duplication and/or segregation.

We conclude that elevated levels of DnaB in an otherwise wild-type cell result in a decrease in the number of initiation events at *oriC* per cell cycle. However, once initiation has occurred, then chromosome duplication and segregation can be completed successfully. In contrast, elevated DnaB levels in the absence of Rep result in failure complete chromosome duplication.

### Defects in replication initiation at or away from *oriC* increase sensitivity to *dnaB* overexpression

Loading of DnaB onto ssDNA at *oriC* occurs via DnaA whilst loading of DnaB away from *oriC* is catalysed by PriA and PriC [6], [13]. Therefore, we tested whether *dnaB* overexpression caused defects in *oriC*-dependent and/or *oriC*-independent replication initiation by screening for synergy between *dnaB* overexpression and replication initiator mutations partially defective in loading of DnaB.

**Fig. 6:**
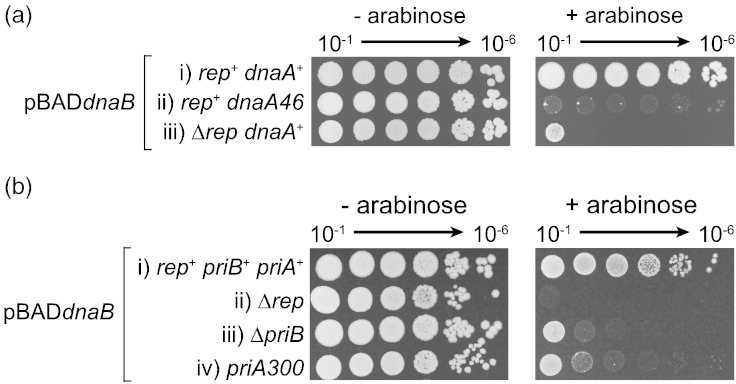
Defects in replication initiation at and away from *oriC* result in hypersensitivity to *dnaB* overexpression. (a) Colony-forming ability of strains containing wild type and temperature-sensitive alleles of *dnaA* together with pBAD*dnaB* (pJGB143) were monitored at the permissive temperature of 30 °C on LB agar containing 0% or 0.2% arabinose. Strains (i)–(iii) were TB28, HB159 and N6577, respectively. (b) Colony-forming ability of the indicated strains containing pBAD*dnaB* (pJGB143) grown on LB agar containing 0% or 0.2% arabinose at 37 °C. Strains (i)–(iv) were TB28, N6577, AM2017 and N5926.

*dnaA46* is a temperature-sensitive allele of the *oriC*-specific replication initiator that can sustain *oriC*-dependent replication initiation and therefore cell division at 30 °C but not at 42 °C. However, even at 30 °C, this allele is not fully functional [43], [44]. We tested *dnaB* overexpression in a *dnaA46* strain at the permissive temperature and found that, although colonies could form, they displayed significant growth defects as compared with *dnaA*^+^ cells (Fig. 6a, compare i and ii).

PriC-dependent replisome reloading is not important in countering the impact of *dnaB* overexpression, as indicated by the lack of hypersensitivity in Δ*priC* cells (Fig. 1D). We could not test Δ*priA* hypersensitivity due to the already poor viability of cells lacking PriA [45]. Therefore, we tested a strain that contains a mutant PriA that can still bind forked DNA structures and load DnaB but that is partially defective in replisome reloading due to the absence of PriA helicase activity [25], [46], [47]. A strain bearing this *priA300* allele displayed hypersensitivity to *dnaB* overexpression (Fig. 6b-iv). This hypersensitivity was not as extreme as that shown by Δ*rep* cells (Fig. 6b, compare ii and iv), but this intermediate growth inhibition could be the result of the partial defect in, as opposed to absence of, PriA-dependent DnaB reloading displayed by *priA300*. Δ*priB* cells also have a partial defect in PriA-directed replisome reloading [48] and were also hypersensitive to *dnaB* overexpression (Fig. 6b-iii).

We conclude that cells with partial defects in either DnaA-dependent replication initiation at *oriC* or PriA-dependent replication reinitiation away from *oriC* display elevated sensitivities to *dnaB* overexpression.

## Discussion

We have demonstrated that overexpression of the replicative helicase DnaB in *E. coli* inhibits growth and that this inhibition can be countered by the helicase loader, DnaC. Overexpression of DnaB impacts upon both DnaA-dependent replication initiation at *oriC* and PriA-dependent replication reinitiation away from *oriC*. Absence of the accessory replicative helicase Rep also sensitises cells to *dnaB* overexpression, an effect that correlates with an increased need for replication reinitiation away from *oriC* in Δ*rep* cells. These findings indicate that elevation of DnaB concentration inhibits both the initiation of chromosome duplication and replication reinitiation after replication forks break down. Therefore, both elevated DnaB, as shown here, and elevated DnaC [1], [2] can inhibit chromosome duplication, highlighting the importance of maintaining appropriate ratios of replicative helicase and helicase loader.

The mechanisms behind the effects of overexpressing either *dnaB* (Fig. 1) or *dnaC* [1], [2] must differ since the causative molecular species formed in each case must be different. Excess DnaC promotes the reassociation of ATP-bound DnaC with ssDNA-bound DnaB and these DnaB–DnaC complexes cannot translocate, inhibiting replication fork movement [10], [11]. How might overproduction of DnaB perturb chromosome metabolism? DnaB-associated growth inhibition is enhanced by defects in initiation of replication at and away from *oriC* (Fig. 6a and b) and by the absence of Rep (Fig. 1, Fig. 3, Fig. 4). Cells lacking Rep display increased replisome pausing and breakdown that elevates the need for replisome reloading away from *oriC* [18], [19], [36], [49]. Mutations that have defects in the initiation of replication (*dnaA46*, *priA300* or *priB* strains) or that increase the need for initiation (Δ*rep*) therefore hypersensitise cells to *dnaB* overexpression. Synergies with Δ*rep*, *priA300* or *priB* could potentially be explained by aberrant binding of excess DnaB onto the chromosome leading to inhibition of the elongation phase of chromosome duplication. Such DnaB binding could promote replisome blockage by forming nucleoprotein barriers and/or catalysing harmful unwinding of DNA structures. However, binding of excess DnaB to double-stranded regions of the chromosome is unlikely since DnaB cannot load onto double-stranded DNA either in the absence or in the presence of DnaC [5], [11]. Furthermore, inhibition of the elongation phase of chromosome duplication would be predicted to cause an increase in origin numbers [24], [50], which is the opposite of what is observed upon *dnaB* overexpression (Fig. 4a and b). Inhibition of elongation is also inconsistent with the synergy observed between *dnaB* overexpression and the partial defect in *oriC*-directed initiation in *dnaA46* cells (Fig. 6a).

The ability of *dnaC* co-overexpression to suppress DnaB toxicity and the need for this co-overexpressed DnaC to interact with DnaB to effect suppression provides an alternative explanation in which excess DnaB inhibits replisome assembly rather than promotes replisome breakdown. Suppression by *dnaC* indicates that it is the formation of DnaB complexes depleted of DnaC that is toxic (Fig. 2). Formation of a DnaB_6_–DnaC_6_ complex induces a conformational transition within the DnaB hexamer that results in a discontinuity within the DnaB ring, allowing entry of ssDNA and hence loading of DnaB onto chromosomes [8]. However, DnaB_6_–DnaC_6_ complexes exist in equilibrium with DnaB hexamers that have fewer than six DnaC monomers bound [51]. Therefore, excess DnaB would reduce the concentration of DnaB_6_–DnaC_6_ complexes and, thus, inhibit loading onto the chromosome. However, whilst it is clear that suppression by *dnaC* requires a functional DnaB–DnaC interaction, evinced by the inability of *dnaC*(*R10P*) *to suppress* DnaB-induced growth defects (Fig. 2b), elevation of DnaC levels could not be detected (Fig. 2c). This lack of detection could be due to rapid degradation of DnaC but it might also reflect the ability to suppress excess DnaB toxicity by substoichiometric levels of DnaC. The cooperative binding of DnaC to DnaB [51] could be one factor in allowing modest overexpression of DnaC to counter DnaB toxicity, facilitating the formation of increased numbers of DnaB_6_–DnaC_6_ complexes even when DnaB remains in excess over DnaC.

A second and not mutually exclusive mechanism might be that DnaB complexes not bound to DNA and depleted of DnaC can interact with DnaG primase and the τ clamper loader subunit [52], [53] whereas DnaB_6_–DnaC_6_ complexes cannot, effectively titrating out other replication enzymes. This titration could inhibit replisome assembly at a step after DnaB loading onto ssDNA. However, regardless of whether inhibition of replisome assembly occurs at the step of DnaB loading or at a later step, this inhibition would be predicted to occur both at *oriC* and away from *oriC* at sites of replication breakdown. Partial inhibition of replisome assembly by elevated DnaB can explain reduced *oriC* initiation events per cell cycle in wild-type cells (Fig. 4a and b) and the hypersensitivity of cells that already bear a partial defect in replisome assembly at (*dnaA46*) and away from *oriC* (*priA300* and Δ*priB*) (Fig. 6). Partial inhibition of replisome reloading might also explain the increased illegitimate recombination caused by *dnaB* overexpression [54] since delayed replisome reloading away from *oriC* might provide sufficient time for inaccurate recombination to occur that is normally outcompeted by accurate repair pathways.

Inhibition of replication initiation also explains the hypersensitivity of Δ*rep* cells (Fig. 1, Fig. 3, Fig. 4, Fig. 5) since increased fork pausing and breakdown in the absence of Rep increases the need for replication reinitiation away from *oriC* [19], [36]. Indeed, the extreme sensitivity of Δ*rep* cells to *dnaB* overexpression as compared with *rep*^+^ cells (Fig. 1, Fig. 4, Fig. 5) provides strong support for a significant increase in the frequency of replisome reinitiation away from *oriC* when Rep is absent. This increased reinitiation is driven primarily by transcription as demonstrated by amelioration of the sensitivity of Δ*rep* cells to *dnaB* overexpression by mutations in RNA polymerase (Fig. 3b).

Elevated DnaB also acts synergistically with defects in PriA-dependent but not PriC-dependent repair (Fig. 1, Fig. 6). The different sensitivities of *priA300 versus priC* cells might be explained by more frequent usage of PriA-directed repair as opposed to PriC that correlates with the severe reduction in viability and increased sensitivity to DNA damaging agents in Δ*priA* but not Δ*priC* cells [45], [55]. Thus, absence of sensitivity to *dnaB* overexpression in Δ*priC* cells again raises questions about whether PriC-catalysed loading of DnaB, independent of PriA, is a physiologically important reaction. However, it remains possible that PriC does play a significant role in replisome reloading *in vivo*. The lack of obvious phenotypes in Δ*priC* cells could reflect an ability of PriA to target damaged forks ordinarily targeted by PriC whereas PriC might not efficiently target substrates normally acted upon by PriA in Δ*priA* cells. The targets and frequencies of use of PriA and PriC *in vivo* remain poorly defined.

In contrast to the bacterial situation, elevating levels of the eukaryotic replicative helicase MCM2-7 would require co-overexpression of multiple genes [56] rather than the single gene found in bacteria. Furthermore, an excess of the eukaryotic replicative helicase is present *in vivo* under normal circumstances and protects against replicative stress by allowing backup origins to be licensed and then used if forks break down [57]. However, our findings demonstrate that an imbalance between just two enzymes required for replisome assembly can result in severe defects in genome duplication. Indeed, overexpression of single MCM subunit genes can contribute to the development of cancer in higher organisms [58], [59], [60], [61]. Our work implies that the phenotypes associated with such overexpression may be dictated by the interacting partners of the subunit whose levels are elevated. Therefore, exquisite coordination in the production of replisome components, especially the replicative helicase and associated enzymes, might be needed regardless of the complexity of the organism.

## Materials and Methods

### Plasmids and strains

Full-length *E. coli dnaB* was amplified from a single colony of *E. coli* that had been resuspended in 50 μl of water and heated at 95 °C for 5 min prior to removal of cell debris by centrifugation. Amplification was performed with a forward primer containing a HindIII site and an NdeI site (*dnaB*_FW in Supplementary Table 1) and a reverse primer containing an XmaI site (*dnaB*_RV). The PCR product was digested with HindIII and XmaI and Klenow treated before ligating into NcoI- and XmaI-digested and Klenow-treated pBAD [18] to form pBAD*dnaB* (pJGB143) bearing kanamycin resistance and an arabinose-inducible *dnaB* gene.

pBAD*dnaBC* was generated by inserting full-length *E. coli dnaC* downstream of *dnaB* into pJGB143 digested with PstI and HindIII, giving pJGB404. For this, *dnaC* was amplified from the genome with a forward primer containing a PstI site plus a 6-bp Shine–Dalgarno sequence up to the Kozak sequence from pBAD24 [62] (*dnaC*_FW.1) and a reverse primer containing a HindIII site (*dnaC*_RV.1). pBAD*dnaC* (pJGB408) is derived from pJGB404 by excising *dnaB* via EcoRI digestion and religation of the 7.1-kb fragment.

*DnaC* was also cloned into pSK(−) as a EcoRI-BamHI fragment so that *dnaC* is inserted in the opposite orientation with respect to the promoter. For this, *dnaC* was amplified using a forward primer with EcoRI and NdeI sites (*dnaC*_FW.2) and a reverse primer with a BamHI site (*dnaC*_RV.2), creating pSK(−)*dnaC*_*inv*_ (pPM202). pJGB412 is pSK(−) encoding *dnaC*(*R10P*)_*inv*_ generated by site-directed mutagenesis of pPM202 using complementary 23-mer forward and reverse primers containing a g29c mismatch flanked by 11 bp of the *dnaC* wild-type sequence [*dnaC*(R10P)_FW and *dnaC*(R10P)_RV]. *dnaC*(*R10P*) from pJGB412 was PCR amplified with the same primers used for pJGB404, digested with EcoRI and HindIII and cloned into pJGB408 digested with EcoRI and HindIII to generate pBAD*dnaC*(*R10P*) (pJGB415). pBAD*dnaBC*(*R10P*) was generated as for pJGB404 except that the mutant *dnaC* from pJGB412 was used as a PCR template, resulting in pJGB418.

All strains used are listed in Supplementary Table 2.

### Spot tests

Strains containing pBAD derivatives were grown in liquid LB containing 5 g l^− 1^ NaCl supplemented with kanamycin (30 μg ml^− 1^) overnight. Growth was performed at 37 °C with the exception of the strains used in Fig. 5a that were grown at 30 °C due to the temperature-sensitive nature of the *dnaA46* allele. Serial dilutions of overnight cultures were made in 56/2 salts from 10^− 1^ to 10^− 6^ and were spotted on LB kanamycin (30 μg ml^− 1^) agar containing 0%, 0.02% or 0.2% arabinose as indicated. The plates were incubated at 37 °C or 30 °C, as indicated, for 16 h.

### SDS polyacrylamide gel electrophoresis

Strains containing pBAD plasmids were grown at 37 °C in LB containing 30 μg ml^− 1^ kanamycin until the *A*_650_ reached 0.4. Ten millilitres of each culture was transferred to a 50-ml Falcon tube and glucose or arabinose was added to each culture to a final concentration of 0.2%. Tubes were incubated at 37 °C with shaking for 2 h then cells were centrifuged for 10 min at 3200*g* at 4 °C, and the supernatant was discarded. Cell pellets were resuspended in lysis buffer [50 mM Tris–Cl (pH 8.5), 150 mM NaCl, 20 mM ethylenediaminetetraacetic acid and 10 mM DTT] and sonicated five times for 10 s. Lysed cells were then centrifuged for 15 min at 16,000*g* at 4 °C and the supernatant was transferred to a new tube and kept on ice overnight. Protein cell extracts were analysed using 12.5% SDS polyacrylamide gels. Protein extract (2 μg) was loaded per lane. NEB Color Prestained Protein Standard, Broad Range (11–245 kDa) and DnaB and DnaC, purified as previously described [63], were run as standards.

### Flow cytometry

Culture samples for flow cytometry were grown in LB plus 30 μg ml^− 1^ kanamycin with shaking at 37 °C until an *A*_650_ of 0.4 was reached. Cultures were then diluted to an *A*_650_ of 0.01 into fresh LB plus 30 μg ml^− 1^ kanamycin and 0.2% arabinose and growth was continued for 2 h. Afterwards, rifampicin and cephalexin were added to 100 μg ml^− 1^ and 15 μg ml^− 1^, respectively, and the cells were grown for another 2 h at 37 °C. Samples were then processed as in Ref. [36] except that flow cytometry was performed on a CyAn ADP Analyser (Beckman Coulter).

### Microscopy

Strains containing pBAD*dnaB* were grown in 10 ml LB supplemented with 30 mg ml^− 1^ kanamycin to an *A*_650_ of 0.4 at 37 °C prior to diluting to an *A*_650_ of 0.01 in 10 ml LB with 30 mg ml^− 1^ kanamycin without and with 0.2% arabinose. Incubation at 37 °C continued for about 2–3 h until the cultures reached *A*_650_ = 0.4, when 1 ml of culture was centrifuged. The pellet was resuspended in 400 μl 56/2 salts. The nucleoids were visualised by staining in 10 μg ml^− 1^ 4′,6-diamidino-2-phenylindole (DAPI) for 5 min and laid on 1% agarose pads containing 56/2 salts. Microscopy was performed on a Zeiss Axioskop2 equipped with a QICAM Fast 1394 camera (QIMAGING) and a DAPI (49) filter set (Zeiss).

The following are the supplementary data related to this article.

Supplementary Table 1 List of PCR primers used in this study. Sequences homologous to target DNA are underlined. Bold letters highlight the mutated base pair in *dnaC*(R10P).

Supplementary Table 2 Strains (TB28 derivatives).

## References

[1] Allen G.C., Kornberg A. (1991). Fine balance in the regulation of DnaB helicase by DnaC protein in replication in *Escherichia coli*. J. Biol. Chem..

[2] Skarstad K., Wold S. (1995). The speed of the *Escherichia coli* fork *in vivo* depends on the DnaB:DnaC ratio. Mol. Microbiol..

[3] McGlynn P. (2013). Helicases at the replication fork. Adv. Exp. Med. Biol..

[4] Itsathitphaisarn O., Wing R.A., Eliason W.K., Wang J., Steitz T.A. (2012). The hexameric helicase DnaB adopts a nonplanar conformation during translocation. Cell.

[5] Kaplan D.L. (2000). The 3′-tail of a forked-duplex sterically determines whether one or two DNA strands pass through the central channel of a replication-fork helicase. J. Mol. Biol..

[6] Mott M.L., Berger J.M. (2007). DNA replication initiation: Mechanisms and regulation in bacteria. Nat. Rev. Microbiol..

[7] Kobori J.A., Kornberg A. (1982). The *Escherichia coli dnaC* gene product. III. Properties of the *dnaB–dnaC* protein complex. J. Biol. Chem..

[8] Arias-Palomo E., O'Shea V.L., Hood I.V., Berger J.M. (2013). The bacterial DnaC helicase loader is a DnaB ring breaker. Cell.

[9] Fang L., Davey M.J., O'Donnell M. (1999). Replisome assembly at *oriC*, the replication origin of *E. coli*, reveals an explanation for initiation sites outside an origin. Mol. Cell.

[10] Davey M.J., Fang L., McInerney P., Georgescu R.E., O'Donnell M. (2002). The DnaC helicase loader is a dual ATP/ADP switch protein. EMBO J..

[11] Gupta M.K., Atkinson J., McGlynn P. (2010). DNA structure specificity conferred on a replicative helicase by its loader. J. Biol. Chem..

[12] Bruning J.G., Howard J.L., McGlynn P. (2014). Accessory replicative helicases and the replication of protein-bound DNA. J. Mol. Biol..

[13] Yeeles J.T., Poli J., Marians K.J., Pasero P. (2013). Rescuing stalled or damaged replication forks. Cold Spring Harb. Perspect. Biol..

[14] Trautinger B.W., Jaktaji R.P., Rusakova E., Lloyd R.G. (2005). RNA polymerase modulators and DNA repair activities resolve conflicts between DNA replication and transcription. Mol. Cell.

[15] Ivessa A.S., Lenzmeier B.A., Bessler J.B., Goudsouzian L.K., Schnakenberg S.L., Zakian V.A. (2003). The *Saccharomyces cerevisiae* helicase Rrm3p facilitates replication past nonhistone protein–DNA complexes. Mol. Cell.

[16] Yeeles J.T., Marians K.J. (2011). The *Escherichia coli* replisome is inherently DNA damage tolerant. Science.

[17] Payne B.T., van Knippenberg I.C., Bell H., Filipe S.R., Sherratt D.J., McGlynn P. (2006). Replication fork blockage by transcription factor–DNA complexes in *Escherichia coli*. Nucleic Acids Res..

[18] Guy C.P., Atkinson J., Gupta M.K., Mahdi A.A., Gwynn E.J., Rudolph C.J. (2009). Rep provides a second motor at the replisome to promote duplication of protein-bound DNA. Mol. Cell.

[19] Boubakri H., de Septenville A.L., Viguera E., Michel B. (2010). The helicases DinG, rep and UvrD cooperate to promote replication across transcription units *in vivo*. EMBO J..

[20] Marians K.J., Hiasa H., Kim D.R., McHenry C.S. (1998). Role of the core DNA polymerase III subunits at the replication fork A is the only subunit required for processive replication. J. Biol. Chem..

[21] McGlynn P., Guy C.P. (2008). Replication forks blocked by protein–DNA complexes have limited stability *in vitro*. J. Mol. Biol..

[22] Mettrick K.A., Grainge I. (2015). Stability of blocked replication forks *in vivo*. Nucleic Acids Res..

[23] Atkinson J., Gupta M.K., McGlynn P. (2011). Interaction of rep and DnaB on DNA. Nucleic Acids Res..

[24] Atkinson J., Gupta M.K., Rudolph C.J., Bell H., Lloyd R.G., McGlynn P. (2011). Localization of an accessory helicase at the replisome is critical in sustaining efficient genome duplication. Nucleic Acids Res..

[25] Sandler S.J. (2000). Multiple genetic pathways for restarting DNA replication forks in *Escherichia coli* K-12. Genetics.

[26] Liu J., Xu L., Sandler S.J., Marians K.J. (1999). Replication fork assembly at recombination intermediates is required for bacterial growth. Proc. Natl. Acad. Sci. U. S. A..

[27] Heller R.C., Marians K.J. (2005). The disposition of nascent strands at stalled replication forks dictates the pathway of replisome loading during restart. Mol. Cell.

[28] Jones J.M., Nakai H. (1999). *Duplex* Opening by primosome protein PriA for replisome assembly on a recombination intermediate. J. Mol. Biol..

[29] McGlynn P., Al-Deib A.A., Liu J., Marians K.J., Lloyd R.G. (1997). The DNA replication protein PriA and the recombination protein RecG bind D-loops. J. Mol. Biol..

[30] Liu J., Nurse P., Marians K.J. (1996). The ordered assembly of the phiX174-type primosome. III. PriB facilitates complex formation between PriA and DnaT. J. Biol. Chem..

[31] Liu J., Marians K.J. (1999). PriA-directed assembly of a primosome on D loop DNA. J. Biol. Chem..

[32] Ng J.Y., Marians K.J. (1996). The ordered assembly of the fX174-type primosome. I. Isolation and identification of intermediate protein–DNA complexes. J. Biol. Chem..

[33] Cadman C.J., Lopper M., Moon P.B., Keck J.L., McGlynn P. (2005). PriB stimulates PriA helicase via an interaction with single-stranded DNA. J. Biol. Chem..

[34] Lopper M., Boonsombat R., Sandler S.J., Keck J.L. (2007). A hand-off mechanism for primosome assembly in replication restart. Mol. Cell.

[35] Heller R.C., Marians K.J. (2005). Unwinding of the nascent lagging strand by rep and PriA enables the direct restart of stalled replication forks. J. Biol. Chem..

[36] Gupta M.K., Guy C.P., Yeeles J.T., Atkinson J., Bell H., Lloyd R.G. (2013). Protein–DNA complexes are the primary sources of replication fork pausing in *Escherichia coli*. Proc. Natl. Acad. Sci. U. S. A..

[37] Ludlam A.V., McNatt M.W., Carr K.M., Kaguni J.M. (2001). Essential amino acids of *Escherichia coli* DnaC protein in an N-terminal domain interact with DnaB helicase. J. Biol. Chem..

[38] McGlynn P., Lloyd R.G. (2000). Modulation of RNA polymerase by (p)ppGpp reveals a RecG-dependent mechanism for replication fork progression. Cell.

[39] Dutta D., Shatalin K., Epshtein V., Gottesman M.E., Nudler E. (2011). Linking RNA polymerase backtracking to genome instability in *E. coli*. Cell.

[40] Trautinger B.W., Lloyd R.G. (2002). Modulation of DNA repair by mutations flanking the DNA channel through RNA polymerase. EMBO J..

[41] Skarstad K., Bernander R., Boye E. (1995). Analysis of DNA replication *in vivo* by flow cytometry. Methods Enzymol..

[42] Lane H.E., Denhardt D.T. (1975). The rep mutation. IV. Slower movement of replication forks in *Escherichia coli rep* strains. J. Mol. Biol..

[43] Lobner-Olesen A., Slominska-Wojewodzka M., Hansen F.G., Marinus M.G. (2008). DnaC inactivation in *Escherichia coli* K-12 induces the SOS response and expression of nucleotide biosynthesis genes. PLoS ONE.

[44] Hinds T., Sandler S.J. (2004). Allele specific synthetic lethality between *priC* and *dnaAts* alleles at the permissive temperature of 30 °C in *E. coli* K-12. BMC Microbiol..

[45] Nurse P., Zavitz K.H., Marians K.J. (1991). Inactivation of the *Escherichia coli priA* DNA replication protein induces the SOS response. J. Bacteriol..

[46] Zavitz K.H., Marians K.J. (1992). ATPase-deficient mutants of the *Escherichia coli* DNA replication protein PriA are capable of catalyzing the assembly of active primosomes. J. Biol. Chem..

[47] Sandler S.J., McCool J.D., Do T.T., Johansen R.U. (2001). PriA mutations that affect PriA–PriC function during replication restart. Mol. Microbiol..

[48] Boonsombat R., Yeh S.P., Milne A., Sandler S.J. (2006). A novel *dnaC* mutation that suppresses *priB rep* mutant phenotypes in *Escherichia coli* K-12. Mol. Microbiol..

[49] Michel B., Ehrlich S.D., Uzest M. (1997). DNA double-strand breaks caused by replication arrest. EMBO J..

[50] Colasanti J., Denhardt D.T. (1987). The *Escherichia coli rep* mutation. X. Consequences of increased and decreased rep protein levels. Mol. Gen. Genet..

[51] Galletto R., Jezewska M.J., Bujalowski W. (2003). Interactions of the *Escherichia coli* DnaB helicase hexamer with the replication factor the DnaC protein. effect of nucleotide cofactors and the ssDNA on protein–protein interactions and the topology of the complex. J. Mol. Biol..

[52] Tougu K., Marians K.J. (1996). The extreme C terminus of primase is required for interaction with DnaB at the replication fork. J. Biol. Chem..

[53] Kim S., Dallmann H.G., McHenry C.S., Marians K.J. (1996). Coupling of a replicative polymerase and helicase: A t-DnaB interaction mediates rapid replication fork movement. Cell.

[54] Yamashita T., Hanada K., Iwasaki M., Yamaguchi H., Ikeda H. (1999). Illegitimate recombination induced by overproduction of DnaB helicase in *Escherichia coli*. J. Bacteriol..

[55] Sandler S.J., Marians K.J., Zavitz K.H., Coutu J., Parent M.A., Clark A.J. (1999). *dnaC* mutations suppress defects in DNA replication- and recombination-associated functions in *priB* and *priC* double mutants in *Escherichia coli* K-12. Mol. Microbiol..

[56] Ilves I., Petojevic T., Pesavento J.J., Botchan M.R. (2010). Activation of the MCM2-7 helicase by association with Cdc45 and GINS proteins. Mol. Cell.

[57] Yekezare M., Gomez-Gonzalez B., Diffley J.F. (2013). Controlling DNA replication origins in response to DNA damage inhibit globally, activate locally. J. Cell Sci..

[58] Ren B., Yu G., Tseng G.C., Cieply K., Gavel T., Nelson J. (2006). MCM7 amplification and overexpression are associated with prostate cancer progression. Oncogene.

[59] Giaginis C., Georgiadou M., Dimakopoulou K., Tsourouflis G., Gatzidou E., Kouraklis G. (2009). Clinical significance of MCM-2 and MCM-5 expression in colon cancer: Association with clinicopathological parameters and tumor proliferative capacity. Dig. Dis. Sci..

[60] Das M., Prasad S.B., Yadav S.S., Govardhan H.B., Pandey L.K., Singh S. (2013). Over expression of minichromosome maintenance genes is clinically correlated to cervical carcinogenesis. PLoS ONE.

[61] Zhong X., Chen X., Guan X., Zhang H., Ma Y., Zhang S. (2015). Overexpression of G9a and MCM7 in oesophageal squamous cell carcinoma is associated with poor prognosis. Histopathology.

[62] Guzman L.M., Belin D., Carson M.J., Beckwith J. (1995). Tight regulation, modulation, and high-level expression by vectors containing the arabinose P_BAD_ promoter. J. Bacteriol..

[63] Marians K.J. (1995). fX174-type primosomal proteins: Purification and assay. Methods Enzymol..

