# Supplementary material for: Overexpression of the Replicative Helicase in Escherichia coli Inhibits Replication Initiation and Replication Fork Reloading
Source: J Mol Biol. 2016 Mar 27;428(6):1068–79. doi: 10.1016/j.jmb.2016.01.018 (PMC4828956; doi:10.1016/j.jmb.2016.01.018)
Supplement: Supplementary Table 1 — List of PCR primers used in this study. Sequences homologous to target DNA are underlined. Bold letters highlight the mutated base pair in dnaC(R10P). [file mmc1.docx]

Supplementary Table 1. List of PCR primers used in this study. Sequences homologous to target DNA are underlined. Bold letters highlight the mutated base pair in *dnaC*(R10P).

| Name | Sequence (5’-3’) |
| --- | --- |
| *dnaB*_FW | GACAAGCTTACATATGGCAGGAAATAAACCCTTCAAC |
| *dnaB*_RV | AGTGGATCCCGGGTTATTATTCGTCGTCGTACTGCG |
| *dnaC*_FW.1 | TGCTGCAG*AGGAGGAATTCACC*ATGAAAAACGTTGGCGACCTG |
| *dnaC*_RV.1 | TCAAAGCTTTTAATACTCTTTACCTGTTACCCGG |
| *dnaC*_FW.2 | CTGAGAATTCATATGAAAAACGTTGGCGACCTG |
| *dnaC*_RV.2 | CGCAGGATCCTTATTAATACTCTTTACCTGTTACCC |
| *dnaC*(R10P)_FW | CCTGATGCAAC**C**CCTGCAAAAAA |
| *dnaC*(R10P)_RV | TTTTTTGCAGG**G**GTTGCATCAGG |
