# Supplementary material for: Overexpression of the Replicative Helicase in Escherichia coli Inhibits Replication Initiation and Replication Fork Reloading
Source: J Mol Biol. 2016 Mar 27;428(6):1068–79. doi: 10.1016/j.jmb.2016.01.018 (PMC4828956; doi:10.1016/j.jmb.2016.01.018)
Supplement: Supplementary Table 2 — Strains (TB28 derivatives). [file mmc2.docx]

Supplementary Table 2. Strains (TB28 derivatives)

| Strain | Genotype | Reference |
| --- | --- | --- |
| TB28 | *∆lacIZYA*::<> | {Bernhardt, 2004 #1834} |
|  |  |  |
| AM2017 | *∆priB*::*dhfr* | {Mahdi, 2012 #19938} |
| AM2158 | *rpoB* (G1260D) | {Guy, 2009 #7397} |
| HB159 | *dnaA46 tna300::*Tn*10* | {Atkinson, 2011 #12235} |
| HB278 | *∆rep::cat rpoB* (G1260D) | {Gupta, 2013 #20621} |
| JGB064 | *rep^+^ <>* | Kn^S^ version of MKG8 generated with pCP20 {Cherepanov, 1995 #6079} |
| JGB066 | *rep∆C33<>* | Kn^S^ version of MKG10 generated with pCP20 {Cherepanov, 1995 #6079} |
| MKG3 | *∆priC*752 | {Gupta, 2013 #20621} |
| MKG8 | *rep^+^ <Kan>* | {Atkinson, 2011 #12235} |
| MKG10 | *rep∆C33<Kan>* | {Atkinson, 2011 #12235} |
| N5925 | *rpoB* (H1244Q) | {Guy, 2009 #7397} |
| N5926 | *priA300* | {Mahdi, 2006 #2133} |
| N6577 | *∆rep::cat* | {Guy, 2009 #7397} |
| N6632 | *∆uvrD::dhfr* | {Guy, 2009 #7397} |
| N7604 | *∆rep::cat rpoB* (H1244Q) | {Gupta, 2013 #20621} |
| SS1076 | *rep-2001 (repK28R)* | {Gupta, 2013 #20621} |
